# Non-healing Wound After Mastopexy: Cautionary Case Report for a Unique Source of a Marjolin’s Ulcer of the Breast

**DOI:** 10.7759/cureus.17504

**Published:** 2021-08-27

**Authors:** Harsh Patel, Yasmina Samaha, Reva Basho, Dhivya R Srinivasa, Alice P Chung

**Affiliations:** 1 Department of Plastic Surgery, University of California, Los Angeles (UCLA), Los Angeles, USA; 2 Department of Plastic and Reconstructive Surgery, Cedars-Sinai Medical Center, Los Angeles, USA; 3 Department of Hematology and Medical Oncology, Cedars-Sinai Medical Center, Los Angeles, USA; 4 Department of Surgery, Cedars-Sinai Medical Center, Los Angeles, USA

**Keywords:** breast surgery, breast cancer, wound management, marjolin's ucler, wound reconstruction

## Abstract

Marjolin’s ulcers (MUs) represent a unique degenerative process that results in malignancy. Classically, sites of previous burns are associated with MU but, in fact, any non-healing wound has been found to be a potential source of degeneration. Malignancies that arise include typically cutaneous squamous cell carcinoma (SCC), but SCC at the site of a previous wound is a more aggressive, lethal variant. This report represents a cautionary case of the management of an open wound and highlights a previously undescribed etiology of an MU in the breast.

## Introduction

The entity of malignant degeneration at the site of a chronic wound, most commonly at the site of a previous burn injury, is referred to as a Marjolin’s ulcer (MU). The eponym was developed after its initial description in the medical literature in 1828 by Dr. Jean Nicholas Marjolin, and the classic triad of nodule formation, induration, and ulceration at a scar site is commonly used to diagnose an MU, especially after a biopsy [1,2]. The literature describes cases of MU arising at the site of previous burns, chronic ulcers (i.e., venous stasis and decubitus), and pressure sores, but here we report the first case of MU of the breast after chronic wounds post-mastopexy for extreme weight loss. Although a previous case report exists of a breast MU [3], this is the first that describes MU in the setting of chronic ulceration at the site of mastopexy incisions. Our patient’s experience should serve as a cautionary tale when managing chronic wounds in patients and illustrates the need for early detection and management to minimize mortality. 

## Case presentation

**Figure 1:**
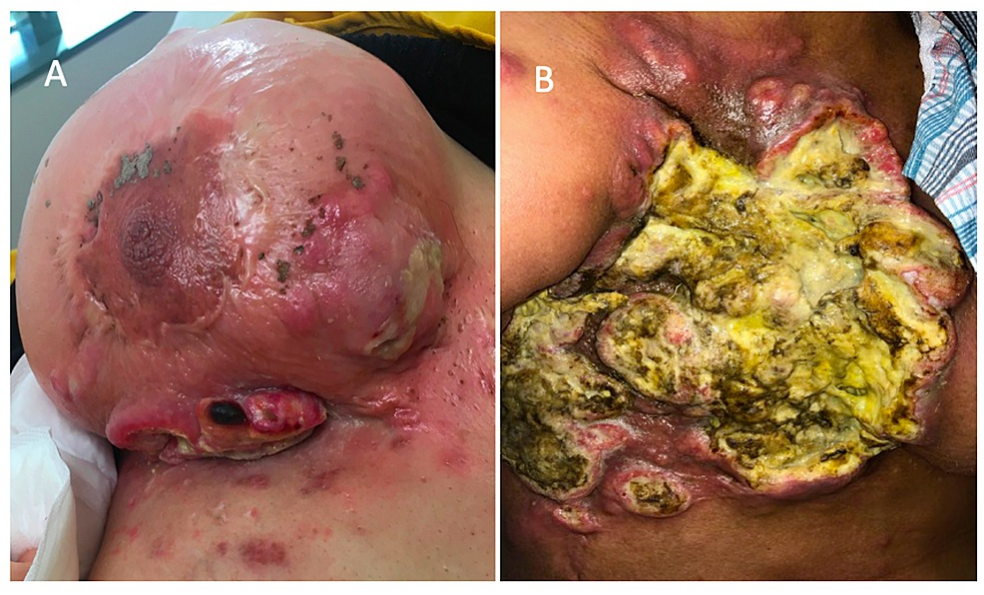
Patient images of cutaneous manifestation. (A) Patient image at presentation to the clinic. (B) Most current image after resection, skin graft, and radiation.

A previously healthy 43-year-old female presented to the surgical oncologist after evaluation by medical oncology, who felt her open wounds made her a poor candidate for chemotherapy. She presented with a large fungating right breast mass (Figure 1). She endorsed a history of a chronic, non-healing right breast wound that began after mastopexy (five years prior) for extreme weight loss (~100 lbs) after consecutive pregnancies. Her contralateral wound healed after reconstruction, but the right breast wound continued to progress despite attempted reconstruction. She noted a small mass at the surgical incision, which grew rapidly in the subsequent months developing into a fungating lesion. Outside hospital biopsy was significant for squamous cell carcinoma (SCC), although the pathologist was unable to differentiate between cutaneous and metaplastic breast SCC. Physical exam confirmed a large fungating lesion of the right breast expanding onto her anterior abdominal wall and axilla with a fixed mass on the chest wall, with multiple palpable axillary nodes on the right. Erythema extended over the entirety of the breast footprint, crossing the midline with satellite lesions on the upper abdominal wall.

**Figure 2:**
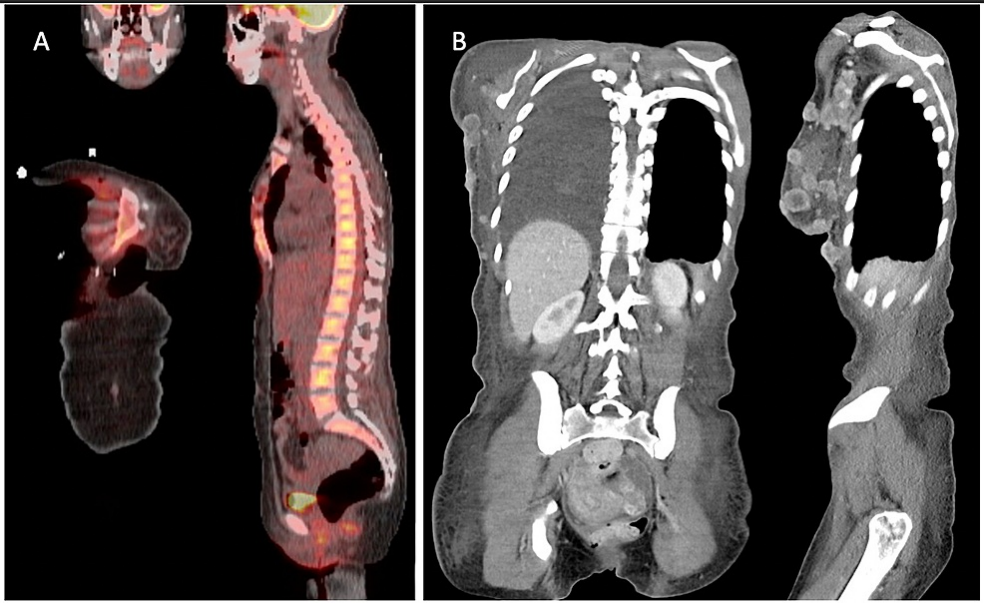
PET imaging on POD1 after initial excision and most recent CT. (A) PET scan after initial excision. Note increased activity along R internal mammary lymph nodes. (B) Most recent CT scan showing extensive subcutaneous disease. PET: positron emission tomography; POD1: postoperative day 1.

She underwent excision with planned immediate reconstruction for wound coverage rather than breast reconstruction. Despite radical excision of her breast, pectoralis major, and level 1/2 lymph nodes, the surgeon felt she had disease adherent to the ribs. Given the extent of the unanticipated disease, the decision was made to delay reconstruction until further staging and resultant pathology. While pathology was pending, the patient underwent positron emission tomography (PET) scan for staging on post-operative day 1 notable for increased activity concerning for malignancy in the right internal mammary lymph node chain and left axially lymph node enlargement (Figure 2). On repeat visits to the operating room to exchange her wound vacuum, the surgeon noted signs of progressive disease, which was confirmed by intraoperative pathology.

Pathology from the initial excision confirmed the diagnosis of squamous cell carcinoma, with a 9.5-cm central mass with extensive involvement of the breast parenchyma and skeletal muscle. Additionally, positive deep surgical margins and four out of 11 positive lymph nodes reaffirmed the decision to delay reconstruction. Lastly, the pathologist echoed a cutaneous origin of SCC disease citing the presence of SCC *in situ* focally at the periphery of the ulcer, the lack of admixed conventional invasion and *in situ* breast carcinoma, and the clinical history of a long-standing ulcer. An attempt at chest wall resection was aborted due to extensive disease on exploration. Critical to note is that surgical manipulation of the tumor bed seemed to trigger a massive proliferation with local extension occurring rapidly. On the two following operating room trips, numerous additional nodules beyond the original margins sprouted in all directions. Therefore, radical excision with intent for curative resection was not possible.

To prevent further delay of non-surgically amenable disease, the patient was offered split-thickness skin grafting over her open wound to allow for prompt radiation. On HD21, she was taken for skin grafting. After debridement, the final wound bed measured 45 x 40 cm. Elevation of the supraclavicular tissue, overlying latissimus skin, medialization of the contralateral breast, and reverse abdominoplasty reduced the area to 20 x 15 cm, which was skin-grafted over. She was discharged on post operative day 4 (HD25) with home health. Despite systemic and local therapy with cemiplimab, the patient had disease progression (Figure 2B) and subsequently succumbed to her disease.

## Discussion

MU represents a unique presentation of malignancy that occurs at the site of a chronic, non-healing wound, previous scar tissue, or site of long-standing inflammation [4]. Most commonly found at the site of a previous burn, 1-2% of burn scars degenerate into malignancy [2]. The duration from injury to malignant degeneration serves as a distinguishing factor among differing types; acute degeneration is defined as within 12 months of injury and chronic as greater than 12 months. SCC is the most common malignancy identified but can include basal cell carcinoma, melanoma, sarcoma, or other types of malignancy [5].

Although diagnosed as SCC, SCC arising from MU behave quite distinctly from non-MU cutaneous SCC. Generally, SCC arising from a chronic wound is more aggressive, with roughly 32% of patients presenting with diffuse disease at the time of diagnosis [2]. Additionally, greater than 27% of patients will have metastatic disease prior to administration of any therapy [6]. Lastly, lymph node disease is more prevalent and confers a grimmer prognosis, with one study citing mortality within two to three years in patients with lymph node-positive disease [2].

Given the aggressive nature of the disease, specifically, the futility of surgical resection with severely delayed presentation, the timing of therapy is essential. Initial local treatment is predicated on wide local excision with negative margins. In cases where this is not feasible, chemotherapy or downstaging radiation is recommended [4,7-9]. Chemotherapy also has a role in the setting of unfavorable prognosis or metastatic disease; the role of adjuvant radiation is debated given the impact it has on local tissue healing, evidenced in our patient [4,7].

## Conclusions

Herein, we present a unique case of MU in the setting of a chronic wound of the breast that arose after the patient underwent mastopexy after weight loss. The novelty of the case resides in the site and cause of the wound that subsequently led to her extensive SCC. It is important to note that she represents the cohort of MU patients that present with extensive disease, in her case due to delayed management of her original wound. This case serves as a cautionary example for the management of persistent wounds with delayed healing to include prompt biopsy. Prognosis hinges on early diagnosis and management.
